# Comparative Performance of Aptima BV/CV-TV and Vaginal Real Time Assays for the Molecular Diagnosis of Bacterial Vaginosis and Vaginitis

**DOI:** 10.3390/diagnostics16193173

**Published:** 2026-09-29

**Authors:** Laura Gozalo Álvarez, Lucía Pérez Rodríguez, Elvira García-Valdecasas García, Ana Fuentes López, Adolfo de Salazar, Federico García

**Affiliations:** 1 Servicio de Microbiología, Hospital Universitario Clínico San Cecilio, 18016 Granada, Spain; lauragozalvarez@gmail.com (L.G.Á.); luciapr1996@gmail.com (L.P.R.);; 2 Instituto de Investigación Biosanitaria IBS.GRANADA, 18012 Granada, Spain; 3 CIBER de Enfermedades Infecciosas (CIBERINFEC), Instituto de Salud Carlos III, 28029 Madrid, Spain

**Keywords:** bacterial vaginosis, vaginitis, molecular diagnosis, nucleic acid amplification tests, sexually transmitted infections

## Abstract

**Background/Objectives**: Direct comparisons between commercial molecular platforms for vaginitis diagnosis are scarce. We compared the Aptima BV and CV/TV assays (Hologic) with the Vaginal Real Time kit (Vircell) for bacterial vaginosis (BV), *Candida* spp. and *Trichomonas vaginalis*, and explored the association between BV and sexually transmitted infection (STI) co-detection. **Methods**: In this prospective single-centre study, 350 vaginal swabs submitted for molecular STI screening were tested on both platforms. Discordances were arbitrated with the Allplex™ Vaginitis Screening assay (Seegene) within a composite reference standard (CRS). Agreement was assessed with Cohen’s κ and positive/negative percentage agreement (PPA/NPA); performance was calculated against the CRS. **Results**: Agreement was substantial for BV (κ = 0.676; PPA 94.8%, NPA 79.1%) and almost perfect for *Candida* spp. (κ = 0.885) and *T. vaginalis* (κ = 0.920), in line with previous molecular-versus-Nugent comparisons. Vircell classified more samples as BV-positive than Aptima (45.4% vs. 33.1%; *p* < 0.001); the Aptima BV positivity rate closely matched the 34% reported by Vipond et al. Against the CRS, Aptima showed higher specificity (98.7%) and Vircell higher sensitivity (98.3%). Agreement fell to 75.9% in women aged ≥45 years. BV positivity was associated with STI co-detection, strongest for Chlamydia trachomatis (OR 8.97, 95% CI 3.26–24.71). **Conclusions**: The two platforms performed equivalently for candidiasis and trichomoniasis but diverged for BV, particularly in older women, reflecting their different classification algorithms rather than analytical quality.

## 1. Introduction

Lower genital tract infections are one of the most frequent reasons for gynaecological consultation in women of reproductive age, with bacterial vaginosis (BV), vulvovaginal candidiasis (VVC) and trichomoniasis accounting for the majority of cases [1,2]. BV is the most common, with rates of 23–29% in the general population [3,4] and is characterised by a vaginal dysbiosis with a decrease in protective *Lactobacillus* spp. and an overgrowth of anaerobes such as *Gardnerella vaginalis*, *Fannyhessea vaginae* (formerly *Atopobium vaginae*, *Prevotella* spp. and *Mobiluncus* spp. [5].

Although often asymptomatic, BV can present with whitish-grey discharge, amine-like odour, vaginal discomfort or dysuria. Together with trichomoniasis, it has been associated with an increased risk of sexually transmitted infections (STI) (HIV, *Chlamydia trachomatis* [CT], *Neisseria gonorrhoeae* [NG], *Mycoplasma genitalium* [MG]), pelvic inflammatory disease and adverse obstetric outcomes such as preterm birth [6,7,8,9]. VVC affects up to 75% of women at some point during their reproductive life, with a growing relevance of non-albicans species [10,11]. These species—particularly *Nakaseomyces glabrata* (formerly *Candida glabrata*) and *Pichia kudriavzevii* (formerly *C. krusei*)—show reduced susceptibility to azoles, so their detection may have therapeutic implications, prompting longer or alternative antifungal regimens rather than standard short-course azole therapy. Accurate and rapid aetiological diagnosis is therefore essential.

Traditional algorithms—Amsel criteria, wet-mount microscopy, Gram stain with Nugent scoring and culture [1,12]—show relevant limitations: interobserver variability in Nugent scoring (up to 20% discordance in duplicate readings) [13], limited sensitivity for *Candida* spp. and *Trichomonas vaginalis*, for which guidelines recommend nucleic acid amplification tests (NAATs) as the reference standard [14,15], longer turnaround times and the inability to detect co-infections [16].

Commercial molecular assays now enable simultaneous, objective and reproducible diagnosis within a few hours [17,18,19,20]. The Aptima BV and Aptima CV/TV assays (Hologic, San Diego, CA, USA), run on the automated Panther platform, rely on transcription-mediated amplification (TMA) and have recently been clinically validated against conventional microscopy and culture in multiple settings [13,21], whereas the Vaginal Real Time kit (Vircell, Granada, Spain) is a CE-IVD multiplex real-time polymerase chain reaction (PCR) with a semi-quantitative four-grade classification (G1–G4). Despite sharing most of their targets, the two platforms differ in amplification technology, detection panels and classification algorithms, which may produce discordant results in clinical practice, and direct comparisons between commercial molecular platforms remain scarce [21,22]. A third CE-IVD platform, the Allplex™ Vaginitis Screening assay (Seegene), was used in this study to arbitrate discordant results. This multiplex real-time PCR provides quantitative detection of the main BV-associated markers—*Lactobacillus* spp., *Gardnerella vaginalis* and *Fannyhessea vaginae*—and classifies samples as normal, intermediate or BV-positive. It has been clinically validated for the diagnosis of bacterial vaginosis [23], which supports its use as an independent reference in the present study.

The aim of the present study was to compare the diagnostic performance of the Aptima BV and Aptima CV/TV assays (Hologic) against the Vaginal Real Time kit (Vircell) for the detection of BV, *Candida* spp. and *T. vaginalis* in vaginal samples from adult women, using the Allplex™ Vaginitis Screening assay (Seegene) as an arbiter for discordant results. As an exploratory analysis, the co-detection of sexually transmitted infections (CT, NG and MG) was also evaluated according to BV status.

## 2. Materials and Methods

### 2.1. Study Design and Population

An observational, prospective, cross-sectional study was carried out at the Microbiology Department of the Hospital Universitario Clínico San Cecilio (Granada, Spain) between November 2025 and March 2026. All vaginal swabs sent to the laboratory for screening of STIs using the Aptima assays on the Panther platform, and for which detection of CT, NG, MG and *T. vaginalis* (TV) was requested, were consecutively included. Both molecular platforms were selected because they are used routinely in our laboratory: the Vaginal Real Time kit for the study of vaginal discharge and the Aptima assays for STI screening. Comparing the two assays already in parallel clinical use reflects a real diagnostic scenario rather than a purpose-built comparison.

From the same primary sample, collected with the Aptima Multitest Swab ( Hologic, Inc., San Diego, CA, USA) in its specific transport medium, the Aptima BV and Aptima CV/TV assays (Hologic) and the Vaginal Real Time kit (Vircell, Granada, Spain) were additionally performed. Samples with discordant results between the two platforms were analysed with a third molecular method, the Allplex™ Vaginitis Screening assay (Seegene, Seoul, Republic of Korea), which was used to arbitrate the discordance.

Patients under 18 years of age and samples with an invalid result in any of the assays compared were excluded, yielding a final study population of 350 samples. All specimens were anonymised surplus material from the routine STI-screening workflow; consequently, no clinical data—including symptom status—were available. Given this sampling frame, a substantial proportion of women were likely asymptomatic or only mildly symptomatic, a point addressed in the study limitations.

### 2.2. Sample Collection and Storage

Samples were obtained by vaginal swabbing with the Aptima Multitest Swab device and transported to the laboratory in their specific medium. Upon receipt, they were processed fresh on the Panther platform (Hologic) with the Aptima CT/NG, Aptima MG, Aptima BV and Aptima CV/TV kits. Subsequently, from the remainder of the primary sample, the Vaginal Real Time assay (Vircell) was performed, including automated nucleic acid extraction on the MagXtract^®^ 3200 system and amplification on the CFX96™ Real-Time PCR Detection System thermocycler (Bio-Rad, Hercules, CA, USA). The eluate obtained after extraction was stored at −80 °C, and samples with discordant results were tested with the Allplex™ Vaginitis Screening assay (Seegene), with subsequent amplification on the same CFX96™ thermocycler (Bio-Rad Laboratories, Inc., Hercules, CA, USA).

### 2.3. Molecular Detection on the Hologic Panther Platform (Aptima BV and Aptima CV/TV)

The Aptima BV and Aptima CV/TV assays were performed on the automated Panther system (Hologic) according to the manufacturer’s instructions. Both assays are based on TMA of specific regions of 16S ribosomal RNA and provide a qualitative result (positive/negative).

The Aptima BV assay detects and relatively quantifies Lactobacillus species associated with normal microbiota (*L. gasseri*, *L. crispatus* and *L. jensenii)*, as well as *G. vaginalis* and *F. vaginae;* the BV result is obtained through a proprietary algorithm that integrates the relative loads of these targets and interprets a positive or negative BV result. The Aptima CV/TV assay detects the *Candida complex (C. albicans*, *C. tropicalis*, *C. parapsilosis* and *C. dubliniensis)*, *Nakaseomyces glabrata* (formerly *C. glabrata*) and *T. vaginalis*.

In addition to the Aptima BV and Aptima CV/TV assays, all samples had been concurrently tested on the Panther platform for the detection of CT, NG (Aptima Combo 2) and MG (Aptima MG) as part of the routine STI screening workflow; these existing STI results were retrieved and analysed post hoc to explore the association between BV status and STI co-detection.

### 2.4. Molecular Detection with the Vaginal Real Time Kit (Vircell)

Nucleic acid extraction and PCR setup were carried out on the MagXtract^®^ 3200 system (Chroma ATE Inc., Taoyuan, Taiwan) following the manufacturer’s instructions, using 300 µL of sample and a final elution volume of 80 µL. Amplification and detection were performed on a CFX96™ Real-Time PCR Detection System thermocycler (Bio-Rad, Hercules, CA, USA). Automatic interpretation of the results was carried out using the Vircom software (Vircell, Granada, Spain).

The Vaginal Real Time kit is a multiplex real-time PCR assay that simultaneously detects eight microorganisms in two reaction mixes: *G. vaginalis*, *Lactobacillus* spp. (*L. crispatus*, *L. jensenii*, *L. iners* and *L. gasseri*), *F. vaginae* and *T. vaginalis* (mix A); and *C. albicans*, *N. glabrata*, *Pichia kudriavzevii* (formerly *C. krusei*) and other Candida species (*C. parapsilosis*/*C. tropicalis*/*C. dubliniensis*) (mix B). The interpretation algorithm classifies samples into four grades according to the balance between *Lactobacillus* spp., *G. vaginalis* and *F. vaginae*: normal microbiota grade 1 (NM G1), normal microbiota grade 2 (NM G2), bacterial vaginosis grade 3 (BV G3) and bacterial vaginosis grade 4 (BV G4). For the comparative analyses, the BV G3 and BV G4 categories were considered positive for BV, and the NM G1 and NM G2 categories were considered negative [22].

### 2.5. Resolution of Discordances Using Allplex™ Vaginitis Screening Assay (Seegene)

Samples with discordant results between Aptima and Vaginal Real Time were analysed with the Allplex™ Vaginitis Screening assay, a multiplex real-time PCR that simultaneously detects *Lactobacillus* spp. (*L. crispatus*, *L. gasseri*, *L. jensenii*), *G. vaginalis*, *F. vaginae*, *Mobiluncus* spp. (*M. mulieris*, *M. curtisii*), *C. albicans*, other Candida species *(C. krusei*, *N. glabrata*, *C. dubliniensis*, *C. parapsilosis*, *C. tropicalis*, *C. lusitaniae*) and *T. vaginalis*. The assay provides quantitative detection of *Lactobacillus* spp., *G. vaginalis* and *F. vaginae*, and includes an automated algorithm for BV classification. Based on the amplification status of these targets, the Seegene Viewer software (v 3.34.003.005) classifies samples as Normal, Intermediate or Positive for BV. For the comparative analyses, samples classified as Positive were considered BV-positive, whereas those classified as Normal or Intermediate were considered BV-negative. The assay was performed using the remnant eluate from the MagXtract^®^ 3200 extraction previously carried out for the Vaginal Real Time assay (Vircell), which had been stored at −80 °C; amplification was performed on the same CFX96™ Real-Time PCR Detection System (Bio-Rad, Hercules, CA, USA). A summary of the microbial targets and main technical characteristics of the three platforms is provided in Table 1 and Table 2.

### 2.6. Statistical Analysis

Bacterial vaginosis lacks a single, universally accepted diagnostic gold standard: Nugent scoring shows interobserver variability and an ambiguous intermediate category, and no molecular assay has been established as an absolute reference, since each platform defines dysbiosis using different targets and algorithms. Because two index assays compared head-to-head cannot adjudicate their own discordances, we applied a composite reference standard (CRS): concordant Aptima and Vaginal Real Time results were taken as the reference, discordant samples were resolved with the Allplex™ Vaginitis Screening assay, and the CRS was defined as the result obtained by at least two of the three platforms. Discordant samples without a valid Allplex result were excluded (*n* = 5 for BV; *n* = 4 for CV/TV). Allplex was chosen as the arbiter because it is an independent, third CE-IVD platform—distinct from the two assays under evaluation—that provides quantitative detection of the main BV-associated markers (*Lactobacillus* spp., *G. vaginalis* and *F. vaginae*) and has been clinically validated for BV diagnosis [23]. All assays were performed and interpreted independently using the manufacturers’ automated software; samples were anonymised, and the Allplex arbitration relied on the automated Seegene Viewer interpretation, so operators had no access to clinical information.

Because the three assays use different Candida groupings, Candida results were collapsed into a binary outcome (positive vs. negative). One sample positive only for *P. kudriavzevii* on the Vaginal Real Time kit was excluded from the Candida analysis, as this target is not detected by Aptima CV/TV. *T. vaginalis* was analysed as an independent binary endpoint.

Sensitivity, specificity, positive predictive value (PPV) and negative predictive value (NPV) were calculated against the CRS, with 95% confidence intervals using the Wilson score method. Inter-assay agreement was assessed with Cohen’s kappa coefficient (κ), interpreted according to Landis and Koch [24]. Differences between paired proportions were assessed with the McNemar test. Odds ratios (OR) with 95% confidence intervals were calculated for the association between BV status and STI co-detection. A two-sided *p*-value <0.05 was considered statistically significant. Statistics and figures were performed using R Studio and Graph Pad Prism V8 software.

### 2.7. Ethical Considerations

This study was approved by the local Research Ethics Committee of Hospital Universitario Clinico San Cecilio. The study used surplus samples submitted to the Microbiology Department for routine clinical diagnosis, which were anonymised prior to inclusion. Due to sample anonymisation, only patient age was available, while clinical data could not be accessed. The study protocol was approved under code SICEIA 2025-000449 (31 March 2025).

## 3. Results

### 3.1. Study Population and Prevalence

A total of 350 vaginal swab samples from adult women attending our hospital between November 2025 and March 2026 were included in the final analysis (Figure 1). The median age was 29 years (IQR 24–38; range 18–85). The distribution by age group was as follows: 18–24 years, 27.1% (95/350); 25–34 years, 37.4% (131/350); 35–44 years, 20.0% (70/350); and ≥45 years, 15.4% (54/350).

The Aptima BV assay classified 116/350 samples (33.1%; 95% CI 28.4–38.2%) as positive for BV; the Aptima CV/TV assay detected *Candida* spp. in 95/350 (27.1%; 95% CI 22.8–32.0%) and *T. vaginalis* in 7/350 (2.0%; 95% CI 1.0–4.1%). The Vaginal Real Time assay classified 159/350 (45.4%; 95% CI 40.3–50.7%) as BV-positive, detected *Candida* spp. in 97/350 (27.7%; 95% CI 23.3–32.6%) and *T. vaginalis* in 6/350 (1.7%; 95% CI 0.8–3.7%) (Table 3).

### 3.2. Inter-Assay Agreement

The two platforms showed substantial agreement for the detection of BV (Cohen’s κ = 0.676; 95% CI 0.597–0.754; overall agreement 84.3%) and almost perfect agreement for the detection of *Candida* spp. (κ = 0.885; 95% CI 0.830–0.940; overall agreement 95.4%) and *T. vaginalis* (κ = 0.920; overall agreement 99.7%) (Table 4). The McNemar test confirmed a statistically significant difference between platforms for BV (χ^2^ = 32.07, *p* < 0.001) but not for *Candida* spp. (*p* = 0.80) or *T. vaginalis.* Using Aptima as the comparator, positive/negative percentage agreement (PPA/NPA) was as follows: BV, 94.8%/79.1%; *Candida* spp., 92.6%/96.5%; and *T. vaginalis*, 85.7%/100%.

For BV, 55 of 350 samples (15.7%) were discordant, with a markedly asymmetric pattern: 49 (89.1%) were Aptima-negative/Vircell-positive—of which 27 (55.1%) were Vircell grade 3 and 22 (44.9%) grade 4—reflecting the systematic tendency of the Vaginal Real Time assay to classify more samples as BV-positive. The Allplex™ Vaginitis Screening assay arbitrated 50 evaluable discordances, confirming the Aptima result in 39 (78.0%) and the Vaginal Real Time result in 11 (22.0%); 5 cases yielded inconclusive results and were excluded from the CRS-based analysis. For *Candida* spp. and *T. vaginalis* combined, 17 of 350 samples (4.9%) were discordant; one additional sample positive exclusively for *P. kudriavzevii* on the Vaginal Real Time assay and 4 cases with inconclusive Allplex results were also excluded.

### 3.3. Diagnostic Performance Against the Composite Reference Standard

Against the CRS (BV prevalence 34.8%, *n* = 345; *Candida* spp. prevalence 26.9%, *n* = 346; *T. vaginalis* prevalence 1.7%, *n* = 350), the two assays showed essentially equivalent performance for *Candida* spp. and *T. vaginalis* combined, both achieving 100% sensitivity, ≥97% specificity, ≥93% PPV and 100% NPV. For BV, however, the two assays differed: the Aptima BV assay achieved a higher specificity (98.7%; 95% CI 96.2–99.5%) and PPV (97.4%; 95% CI 92.6–99.1%), while the Vaginal Real Time assay achieved a slightly higher sensitivity (98.3%; 95% CI 94.1–99.5%) and NPV (98.9%; 95% CI 96.2–99.7%), with markedly lower specificity (83.6%; 95% CI 78.2–87.8%) and PPV (76.1%; 95% CI 68.8–82.2%) (Table 5). The two platforms therefore reflect complementary diagnostic profiles for BV: a binary, specificity-oriented algorithm (Aptima) and a four-grade, sensitivity-oriented classification (Vircell).

Species-level comparison for *Candida* spp. was not feasible because of the heterogeneous grouping schemes of the two platforms—the Aptima CV/TV assay reports four species jointly as the *Candida* spp. while the Vaginal Real Time kit reports *C. albicans*, *N. glabrata* and *P. kudriavzevii* individually (see Table 1). Given the very low prevalence of trichomoniasis in our population (1.7%), the study was underpowered for this outcome, although the perfect concordance between platforms on the six CRS-positive samples is reassuring.

Mixed infections were detected by the Aptima panel in 32/350 samples (9.1%) and by the Vaginal Real Time assay in 43/350 samples (12.3%). The most common combination on both platforms was BV plus *Candida* spp. (29 and 39 samples, respectively). Discordant mixed-infection results between the two platforms were also arbitrated with the Allplex assay, applying the same criterion used for the individual targets. 

### 3.4. Subgroup Analysis by Age

Inter-assay agreement for BV varied with age: it reached 90.0% in the 35–44-year group but dropped to 75.9% in women aged ≥45 years. Notably, all 13 discordant cases in the ≥45 group were Aptima-negative/Vircell-positive, with the Vaginal Real Time assay yielding a BV positivity rate of 55.6% in this subgroup compared with 31.5% for Aptima (Δ +24.1 percentage points). This pattern contrasted with the more balanced discordance distribution observed in younger groups (Δ ranging from +7.1 to +12.6 percentage points) and is consistent with previous reports of reduced specificity of molecular BV assays in older women, attributable to age-related shifts in vaginal microbiota composition—although in our study this age group consisted of only 15.4% of the entire cohort

### 3.5. Exploratory Analysis: STI Co-Detection

In the exploratory analysis of STI co-detection, the overall prevalence of any STI was 9.4% (33/350; CT 6.9%, NG 1.7%, MG 2.7% [9/333; 17 invalid results excluded]), with the highest rates in the 18–24-year group (any STI 15.8%) and a progressive decrease with age (Figure 2A). A positive BV result was associated with a higher prevalence of STI co-detection: the odds of any STI in women with a positive Aptima BV result was approximately fivefold that of BV-negative women (OR 5.54, 95% CI 2.54–12.09), with the strongest effect observed for CT (OR 8.97, 95% CI 3.26–24.71); the Vaginal Real Time assay showed the same direction of effect with smaller magnitude (OR 3.60, 95% CI 1.62–7.98) (Figure 2B).

## 4. Discussion

Our findings show that strong overall agreement between molecular assays can mask clinically relevant differences in pathogen- or syndrome-specific classification. While concordance was robust for *Candida* spp. and *T. vaginalis*, BV detection was notably influenced by the platform used, underscoring the need to interpret molecular BV results in the light of assay design, target selection, diagnostic thresholds, and in the context of the patient’s signs and symptoms.

When benchmarked against the CRS, the Aptima BV assay achieved a markedly higher specificity (98.7%) and PPV (97.4%) than the Vaginal Real Time assay (83.6% and 76.1%, respectively), at the cost of a marginally lower sensitivity (93.3% vs. 98.3%). For the combined CV/TV target, both assays performed almost identically (sensitivity 100%, specificity ≥ 97%, PPV ≥ 93%, NPV 100%). A novel observation of our study was the age-dependent divergence in BV agreement: in women aged ≥45 years, concordance fell to 75.9% and every one of the 13 discordant cases was Aptima-negative/Vircell-positive, with the Vaginal Real Time kit yielding a BV positivity rate of 55.6% in this subgroup, compared with 31.5% for Aptima (Δ +24.1 percentage points). In an exploratory analysis, a positive BV result was associated with a higher rate of STI co-detection, particularly for *C. trachomatis*.

Our results align with the scarce literature directly comparing commercial molecular platforms for the diagnosis of vaginitis. The 84.3% inter-assay agreement that we observed for BV is comparable to the 87.7% reported by Vieira-Baptista et al. for the Allplex™ Vaginitis assay against Nugent scoring (κ = 0.69) [23] and to the 92% agreement reported by Amor et al. for the same Vaginal Real Time kit against Nugent in a cohort from our own centre (κ = 0.82) [22]. The slightly lower κ value in our study (0.676) reflects an intrinsic feature of head-to-head molecular comparisons: both platforms are highly sensitive, and disagreements naturally concentrate in the “grey zone” of intermediate dysbiosis, where a binary algorithm (Aptima) and a four-grade gradient (Vircell) are bound to diverge. The asymmetric pattern of discordance we observed—predominantly Aptima-negative/Vircell-positive—is fully consistent with Amor et al., who reported that 117 of 193 (60.6%) Nugent-intermediate samples were classified as BV by the Vaginal Real Time kit [22]. Two complementary explanations are plausible: the Vircell algorithm captures samples with reduced but still detectable *Lactobacillus*, which a strict binary algorithm would classify as normal microbiota; and at least some of these calls likely represent genuine early dysbiosis rather than false positives—a hypothesis supported by the recognised concept of molecular BV [5]. Consistent with this, grade 3 predominated among the discordances (55.1%); nevertheless, the substantial proportion classified as grade 4 (44.9%) indicates that the disagreement was not confined to borderline dysbiosis and is also compatible with a degree of over-classification by the gradient-based algorithm. For the Aptima platform, our specificity (98.7%) and PPV (97.4%) are in line with the recent multicentre validation by Elvy et al. [13], reinforcing the consistency of TMA-based BV diagnosis across geographically diverse populations.

Our findings are further reinforced by the recent UK multicentre evaluation by Vipond et al. (*n* = 2152 women in primary care), which compared the Aptima BV and CV/TV assays against the Hay/Ison criteria as standard of care [21]. They reported a sensitivity of 98.8% and a specificity of 77.7% for the Aptima BV assay and, notably, a BV positivity rate of 34.0%—almost identical to the 33.1% observed in our cohort. Their lower specificity against Hay/Ison microscopy (77.7%, versus the 98.7% that we obtained against the CRS) is largely attributable to the limited sensitivity of microscopy-based scoring rather than to a true loss of test performance: their discrepancy analysis using independent quantitative PCR showed that most Aptima-positive/microscopy-negative samples had an intermediate microbial profile, suggesting that the molecular assay detects emerging dysbiosis before it becomes microscopically evident [21]. This is consistent with our own data, in which the asymmetric Aptima-negative/Vircell-positive discordances likely reflect the same continuum from normal microbiota to overt BV, captured at different thresholds by a binary algorithm (Aptima) and a gradient-based classification (Vircell). This divergence reflects the different design of the two assays rather than a difference in analytical quality. Aptima integrates the relative loads of *Lactobacillus* spp., *G. vaginalis* and *F. vaginae* into a single binary output, whereas the Vaginal Real Time assay applies a semi-quantitative four-grade classification (G1–G4). Because the two algorithms weight and threshold the same targets differently, they diverge chiefly in the intermediate range, where the graded algorithm assigns more samples to the BV-positive categories (G3–G4), explaining the predominantly Aptima-negative/Vircell-positive discordances.

Our age-stratified analysis contributes information not previously reported in head-to-head comparisons of commercial molecular platforms. The almost complete absence of Aptima-positive/Vircell-negative discordances in women aged ≥45 years, together with the near-doubling of the Vircell BV positivity rate in this subgroup, parallels the previously described limitations of molecular BV diagnosis in older women; Vieira-Baptista et al. reported a marked drop in specificity (to 28.9%) in their postmenopausal subset, attributable to age-related *Lactobacillus* depletion that mimics dysbiosis [23]. This pattern is compatible with age- and menopause-related changes in the vaginal ecosystem—declining *Lactobacillus* abundance, rising vaginal pH and a shift towards a more diverse, anaerobe-containing microbiota—which can be misclassified as molecular dysbiosis. A sensitivity-oriented graded algorithm (Vircell) may therefore classify more of these samples as BV-positive, whereas a *Lactobacillus*-weighted binary algorithm (Aptima) behaves more conservatively and may be less prone to false positives driven by atrophic vaginitis. This mechanism is the most plausible explanation for the reduced inter-assay agreement and the predominantly Aptima-negative/Vircell-positive discordances in this subgroup, although it should be confirmed in larger cohorts with documented menopausal status.

For *Candida* spp., the almost perfect agreement we observed (κ = 0.885) and the 100% sensitivity of both assays against the CRS are consistent with previous validations of each platform: Amor et al. reported a sensitivity of 96.0% and a specificity of 98.4% for the Vaginal Real Time kit against culture plus MALDI-TOF MS [22], and Vipond et al. reported similar figures for the Aptima CV/TV assay against culture (sensitivity 94.9%, specificity 88.3%; positivity rate 31.3% vs. 27.1% in our cohort) [21]. Species-level comparison between the two platforms was not feasible, however, given their heterogeneous grouping schemes—a real-world limitation for laboratories selecting a platform according to the local epidemiology of non-albicans species [10,11]. Importantly, molecular detection of *Candida* spp. indicates the presence of yeast DNA but does not by itself establish vulvovaginal candidiasis, since *Candida* colonisation is common in asymptomatic women; in the absence of clinical data, our results should therefore be interpreted as organism detection rather than as a diagnosis of symptomatic infection.

The exploratory association observed between Aptima BV positivity and STI co-detection—particularly for CT (OR 8.97, 95% CI 3.26–24.71)—is broadly consistent with previous reports based on Nugent scoring [7,8] and supports the biological plausibility of enhanced epithelial susceptibility to STI acquisition in the dysbiotic vagina [25,26]. The stronger BV–STI association observed with Aptima than with the Vaginal Real Time assay is consistent with the latter classifying a broader set of samples as BV-positive, thereby diluting the association; this supports the interpretation that the graded algorithm captures a wider dysbiosis spectrum, at the cost of specificity for the more established dysbiosis most strongly linked to STI co-detection.

The principal strengths of our study are its head-to-head design, comparing two CE-IVD molecular platforms on the same set of clinical samples; the relatively large sample size (*n* = 350) compared with previously published platform comparisons; and the use of an independent third molecular assay (Allplex™ Vaginitis Screening) to arbitrate discordant cases. The availability of parallel STI screening data also enabled an exploratory assessment of the BV–STI association in the same cohort.

Some limitations should be acknowledged, although several design features mitigate their impact. The use of anonymised routine samples precluded access to relevant clinical variables—including symptoms, menopausal status, contraceptive use, sexual history and pregnancy—which limits the interpretation of the subgroup findings, particularly the age-related divergence in BV agreement. Relatedly, because specimens originated from an STI-screening workflow rather than from a symptom-driven vaginitis work-up, the cohort may be enriched for asymptomatic or screening-context women and may not fully represent patients presenting with vaginitis symptoms. As neither BV nor Candida screening is recommended in asymptomatic women, a positive molecular result in the absence of symptoms does not by itself establish clinical disease or warrant treatment, and this should be considered when extrapolating our estimates to symptomatic populations.

An additional preanalytical limitation is that all assays were performed from the same primary specimen collected in the Aptima Multitest Swab transport medium (Hologic). While this medium is validated for the Aptima assays, its use with the Allplex™ Vaginitis Screening assay (Seegene) is off-label, as the manufacturer does not include this collection device among its validated specimen types; for the Vaginal Real Time assay (Vircell), the manufacturer’s instructions do not specify this transport medium. Although all three assays yielded valid amplification results, a potential impact of the transport medium on extraction efficiency or analytical sensitivity cannot be fully excluded.

For BV, the absence of a universally accepted molecular reference standard remains the main limitation. The composite reference standard we used was arbitrated by an independent third molecular assay; however, because the Allplex™ assay applies a quantitative, threshold-based classification conceptually closer to the binary Aptima algorithm than to the four-grade Vaginal Real Time interpretation, it may have favoured Aptima in discordant BV samples and underestimated the potential relevance of gradient-based classifications, including early or intermediate dysbiosis. The higher specificity attributed to Aptima should therefore be interpreted as relative rather than absolute. The lack of concurrent Nugent or Hay/Ison scoring further limits comparison with microscopy-based definitions, and 16S rRNA gene sequencing of discordant samples would provide a more taxonomically resolved arbiter in future studies, particularly in the intermediate dysbiosis zone and in older women with age-related *Lactobacillus* depletion.

Finally, species-level comparison for *Candida* spp. was not feasible because of the heterogeneous grouping schemes used by the two platforms, and the very low prevalence of *T. vaginalis* limited the precision of performance estimates for this target, although the complete concordance across the six CRS-positive samples is reassuring. Notwithstanding these limitations, the head-to-head comparison of two CE-IVD molecular platforms on the same clinical specimens, together with a relatively large sample size for this type of platform evaluation, strengthens the internal validity of the analytical comparisons. A SWOT analysis summarising the strengths, weaknesses, opportunities and threats of molecular testing for BV and vaginitis is presented in Table 6.

In summary, our head-to-head comparison of two CE-IVD molecular platforms, the Aptima BV/CV-TV (Hologic) and Vaginal Real Time (Vircell) assays, showed substantial concordance for the diagnosis of BV, with discordant results overly aligned with Aptima, almost-perfect for vulvovaginal candidiasis and trichomoniasis, and equivalent diagnostic performance against a CRS. The most notable exception was BV in women aged ≥45 years, in whom the Vaginal Real Time kit systematically classified more samples as positive. Our secondary analysis evidenced a robust association between molecular BV positivity and STI co-detection, particularly for *C. trachomatis* in young women, supporting the integration of molecular BV diagnosis into a broader reflex-testing strategy for the prevention and early detection of STI.

## Figures and Tables

**Figure 1:**
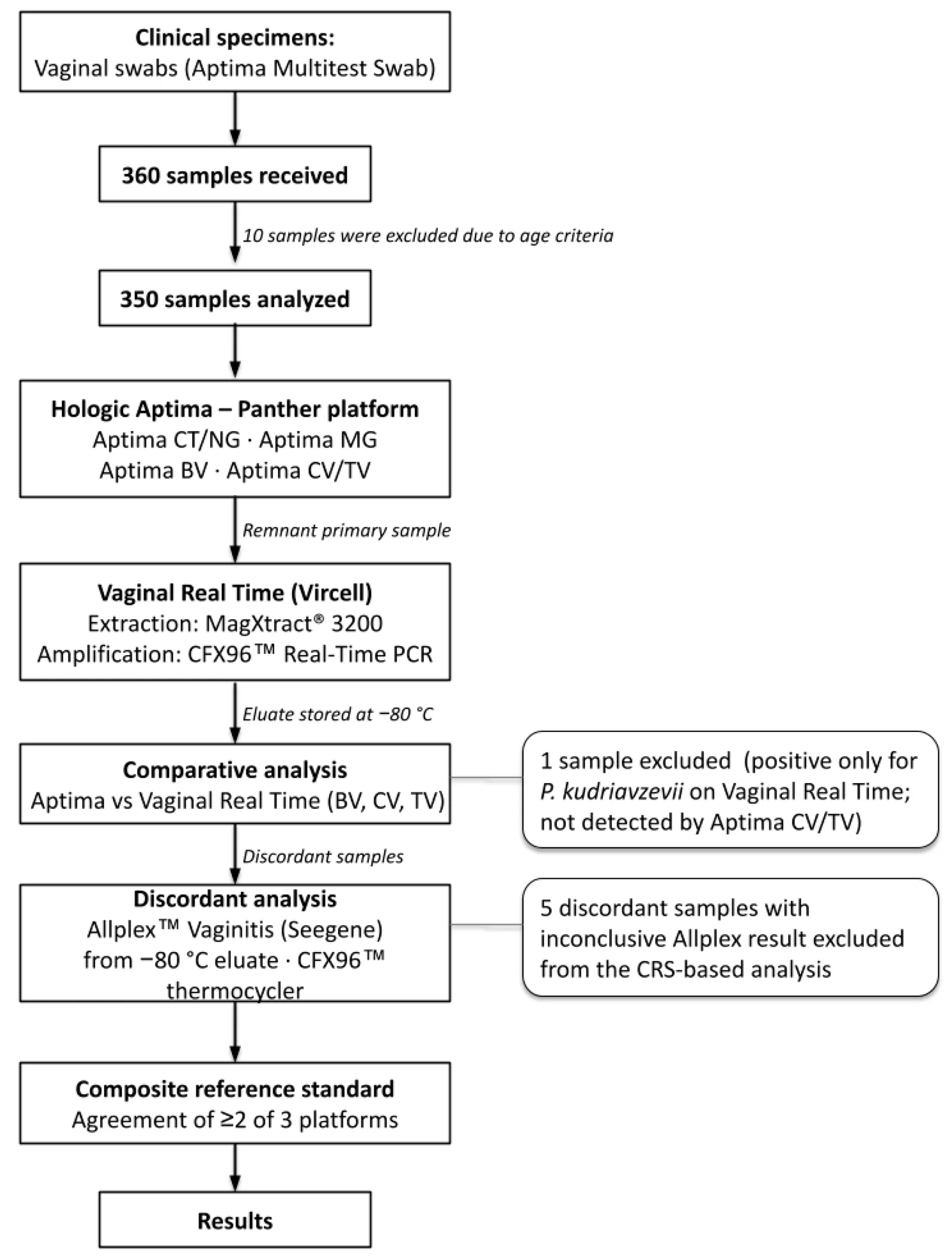
Flow diagram of the study workflow. Vaginal swabs submitted for STI screening at Hospital Universitario Clínico San Cecilio (Granada, Spain) between November 2025 and March 2026 were included. One sample positive only for *P. kudriavzevii* on the Vaginal Real Time kit was excluded from the *Candida* analysis (target not detected by Aptima CV/TV), and 5 discordant samples with an inconclusive Allplex result were excluded from the CRS-based analysis. CRS, composite reference standard.

**Figure 2:**
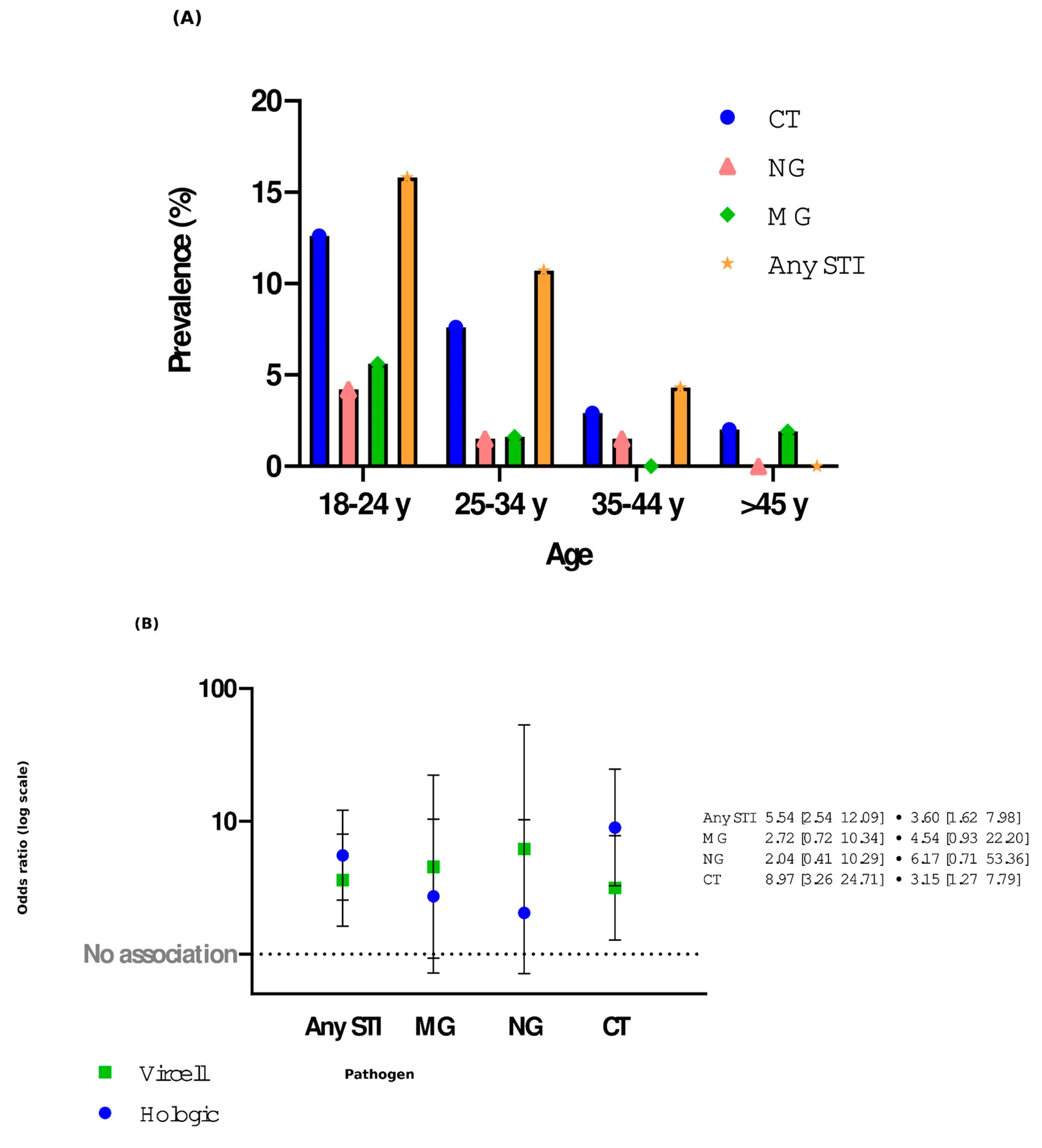
Sexually transmitted infection (STI) co-detection in the vaginal cohort (*n* = 350). (**A**) Prevalence of each STI by age group. (**B**) Odds ratios (with 95% confidence intervals) for STI co-detection in BV-positive versus BV-negative women, shown for each platform. Abbreviations: STI, sexually transmitted infection; CT, *Chlamydia trachomatis*; NG, *Neisseria gonorrhoeae*; MG, *Mycoplasma genitalium*; BV, bacterial vaginosis.

**Table 1:** Comparison of the two molecular platforms evaluated (Aptima BV/CV-TV, Hologic; and Vaginal Real Time, Vircell). Main technical and diagnostic characteristics of the Aptima BV/CV-TV (Hologic) and Vaginal Real Time (Vircell) assays against the composite reference standard. Turnaround time was comparable between platforms.

Feature	Aptima BV/CV-TV (Hologic)	Vaginal Real Time (Vircell)
Amplification technology	Transcription-mediated amplification (TMA)	Multiplex real-time PCR
BV targets	*Lactobacillus* spp., *G. vaginalis*, *F. vaginae* (algorithm-based)	*Lactobacillus* spp. (incl. *L. iners*), *G. vaginalis*, *F. vaginae*
BV result format	Binary (positive/negative)	Four-grade classification (NM G1–G2/BV G3–G4)
Candida targets	Candida complex + *N. glabrata*; *T. vaginalis*	*C. albicans*, *N. glabrata*, *P. kudriavzevii*, other Candida
Performance vs. CRS (BV)	Higher specificity/PPV	Higher sensitivity/NPV
Main advantage	Specificity; STI co-testing on the same specimen	Sensitivity; graded dysbiosis; broader Candida panel
Main limitation	Binary BV output; grouped Candida	Lower BV specificity, especially in older women
Turnaround time/workflow	Easy to perform	Easy to perform

**Table 2:** Comparison of microbial targets and technical characteristics of the three molecular platforms evaluated. Comparison of amplification technology, bacterial vaginosis (BV) interpretation algorithms, target microorganisms, and quantification approaches of the Aptima BV/CV-TV assays (Hologic), Vaginal Real Time assay (Vircell), and Allplex™ Vaginitis Screening assay (Seegene). BV-associated microorganisms, Candida species, and *Trichomonas vaginalis* (TV) targets included in each platform are shown.

Category/Target	Hologic Aptima (BV + CV/TV)	Vircell Vaginal Real Time	Seegene Allplex™ Vaginitis
Amplification	TMA (rRNA and RNA)	qPCR	qPCR
Quantification of bacterial targets	Relative (algorithm)	Semi-quantitative	Quantitative
BV result interpretation	Positive/Negative	4 grades (NM G1, NM G2, BV G3, BV G4)	Normal/Intermediate/Positive
**Lactobacillus species**			
*L. crispatus*	✓	✓	✓
*L. gasseri*	✓	✓	✓
*L. jensenii*	✓	✓	✓
*L. iners*	x	✓	x
**BV–associated bacteria**			
*Gardnerella vaginalis*	✓	✓	✓
*Fannyhessea vaginae*	✓	✓	✓
*Mobiluncus mulieris*	x	x	✓
*Mobiluncus curtisii*	x	x	✓
**Candida/yeasts**			
*C. albicans*	✓ (grouped)	✓	✓
*C. tropicalis*	✓ (grouped)	✓ (grouped)	✓ (grouped)
*C. parapsilosis*	✓ (grouped)	✓ (grouped)	✓ (grouped)
*C. dubliniensis*	✓ (grouped)	✓ (grouped)	✓ (grouped)
*Nakaseomyces glabrata*	✓	✓	✓ (grouped)
*Pichia kudriavzevii*	—	✓	✓ (grouped)
*C. lusitaniae*	—	—	✓ (grouped)
**Protozoa**			
*Trichomonas vaginalis*	✓	✓	✓

**Table 3:** Overall prevalence of bacterial vaginosis, *Candida* spp. and *T. vaginalis* detected by each molecular platform (*n* = 350).

Target	Aptima *n* (% [95% CI])	Vaginal Real Time *n* (% [95% CI])
BV	116 (33.1% [28.4–38.2])	159 (45.4% [40.3–50.7])
*Candida* spp.	95 (27.1% [22.8–32.0])	97 (27.7% [23.3–32.6])
*T. vaginalis*	7 (2.0% [1.0–4.1])	6 (1.7% [0.8–3.7])

**Table 4:** Inter-assay agreement between Aptima and Vaginal Real Time assays for the detection of bacterial vaginosis, *Candida* spp., and *T. vaginalis* (*n* = 350). Contingency tables showing concordant and discordant results between platforms. Agreement was assessed using Cohen’s kappa coefficient (κ), overall percentage agreement, and McNemar’s test for paired proportions.

	Vaginal Real Time +	Vaginal Real Time −	Total
**BV**			
**Aptima +**	110	6	116
**Aptima −**	49	185	234
**Total**	159	191	350
Cohen’s κ = 0.676 (95% CI 0.597–0.754); overall agreement 84.3%; McNemar χ^2^ = 32.07, *p* < 0.001.			
***Candida *** **spp.**			
**Aptima +**	88	7	95
**Aptima −**	9	246	255
**Total**	97	253	350
Cohen’s κ = 0.885 (95% CI 0.830–0.940); overall agreement 95.4%; McNemar *p* = 0.80.			
***T. vaginalis***			
**Aptima +**	6	1	7
**Aptima −**	0	343	343

**Table 5:** Diagnostic performance of Aptima and Vaginal Real Time assays against the composite reference standard. Sensitivity, specificity, positive predictive value (PPV), and negative predictive value (NPV) for bacterial vaginosis and Candida/*Trichomonas* detection are presented with 95% confidence intervals. The composite reference standard was defined by concordance between at least two of the three molecular platforms evaluated.

Assay	Sensitivity (95% CI)	Specificity (95% CI)	PPV (95% CI)	NPV (95% CI)
**Aptima BV**	93.3% (87.4–96.6)	98.7% (96.2–99.5)	97.4% (92.6–99.1)	96.5% (93.3–98.2)
**Vaginal Real Time BV**	98.3% (94.1–99.5)	-	76.1% (68.8–82.2)	98.9% (96.2–99.7)
**Aptima CV/TV**	100% (96.0–100)	97.2% (94.4–98.7)	93.0% (86.3–96.6)	100% (98.5–100)
**Vaginal Real Time CV/TV**	100% (96.0–100)	97.6% (94.9–98.9)	93.9% (87.4–97.2)	-

**Table 6:** SWOT analysis of molecular testing for bacterial vaginosis and vaginitis, based on the two CE-IVD platforms evaluated. The analysis summarises the internal strengths and weaknesses and the external opportunities and threats of molecular diagnosis of bacterial vaginosis and vaginitis, drawing on the head-to-head comparison of the Aptima BV/CV-TV (Hologic) and Vaginal Real Time (Vircell) assays. Strengths and weaknesses refer to intrinsic features of the molecular approach and of the assays themselves, whereas opportunities and threats refer to external factors affecting their implementation in clinical practice.

**Strengths** Objective, reproducible and (semi-)automated detection within hours Simultaneous multi-target coverage Higher sensitivity than microscopy for *Candida* spp. and *T. vaginalis.*	**Weaknesses** No universally accepted molecular reference standard for BV Heterogeneous *Candida* groupings preclude species-level comparison Molecular detection does not equal clinical disease Reduced specificity in women aged ≥45 years.
**Opportunities** Reflex STI testing from the same specimen Standardisation of molecular BV criteria Integration into screening and diagnostic pathways.	**Threats** Over-diagnosis and over-treatment of asymptomatic colonisation Cost Platform-dependent classification that limits comparability across laboratories.

## Data Availability

The data presented in this study are available on request from the corresponding author. The data are not publicly available because they derive from anonymised clinical samples processed for routine diagnosis.

## Abbreviations

BV, bacterial vaginosis; VVC, vulvovaginal candidiasis; STI, sexually transmitted infection; CT, Chlamydia trachomatis; NG, Neisseria gonorrhoeae; MG, Mycoplasma genitalium; NAAT, nucleic acid amplification test; PCR, polymerase chain reaction; TMA, transcription-mediated amplification; CRS, composite reference standard; PPV, positive predictive value; NPV, negative predictive value; PPA, positive percentage agreement; NPA, negative percentage agreement; CI, confidence interval; OR, odds ratio; CE-IVD, Conformité Européenne–in vitro diagnostic.
